# Identifying dispensing errors in pharmacies in a medical science school in Trinidad and Tobago

**DOI:** 10.1186/s40545-020-00263-x

**Published:** 2020-10-08

**Authors:** Sandeep Maharaj, Adrian Brahim, Horry Brown, Danielle Budraj, Vatalie Caesar, Anyse Calder, Deisha Carr, Dion Castillo, Kevin Cedeno, Manthan D. Janodia

**Affiliations:** 1 School of Pharmacy, The University of the West Indies, St Augustine Campus, Trinidad and Tobago; 2 Department of Pharmacy Management, Manipal College of Pharmaceutical Sciences, Manipal Academy of Higher Education, Manipal, Karnataka 576104 India

**Keywords:** Dispensing errors, Pharmacy, Medical school, Trinidad and Tobago

## Abstract

**Background:**

A dispensing error can be defined as an inconsistency between the drug prescribed and drug dispensed to a patient. These errors can lead to ineffective and sometimes unwanted pharmaceutical outcomes. Dispensing errors can be harmful or even fatal to patients.

**Case presentation:**

The objective to this study was (a) to determine the types and frequency of dispensing errors at the Eric Williams Medical Sciences Complex (EWMSC), (b) to explore the reasons for the occurrence of dispensing errors, and (c) to make suitable recommendations for their prevention. An observational study for a period of 2 weeks was carried out at various in- and outpatient departments of the EWMSC. The observations were carried out during 7:00 am to 3:00 pm. Dispensing errors identified during this period were recorded and analyzed.

**Results:**

Sixty-eight errors were identified in the adult outpatient pharmacy of the EWMSC; 19 errors in the pediatric outpatient pharmacy, whereas 22 errors were found in inpatient pharmacy. The most common plausible causes for the dispensing errors include high workload, failure to verify patient information, incorrect data in the pharmacy’s record system, inadequate notes made by pharmacists during prior patient visit, and in a few cases, uncomfortable working conditions.

**Conclusion:**

Dispensing errors were encountered in 2.1% of all the prescriptions filled at the EWMSC pharmacies. The factors which influenced these dispensing errors include but are not limited to a heavy workload, distractions, failure to verify patient information, and uncomfortable working conditions.

## Introduction

In recent times, research on dispensing errors and its impact on patients has increased along with growing interest in the role of automation and computerization in preventing such errors [1, 2]. It is of significant importance to understand and record the types, causes, and frequency of dispensing errors that occur locally and corrective actions taken to minimize such errors. Dispensing errors and its potential harmful effects are well documented in existing literature [1–3]. However, very little research on dispensing errors and its negative effect on patients is conducted in the Caribbean region.

A dispensing error can be defined as an inconsistency between the dispensing of medication to the patient against medications prescribed [3]. These errors can include dispensing of medication with ineffective pharmaceutical outcomes [4]. The dispensing errors include but are not limited to the dispensing of the incorrect drug, incorrect dose strength and frequency, and drug dispensed to wrong patient.

Dispensing errors may cause undue distress and suffering to patients. Some factors leading to dispensing errors include high workload of pharmacists, brands/drugs with phonetic similarity, interruptions and distractions in the dispensing process, and an inability to understand doctor’s handwriting [5].

Due to significant danger involved in dispensing errors, it is very important that their occurrence is minimized and efforts are made to prevent it by identifying the most common causes of such occurrences. This information can then be used in the facilitation and planning of the improvement of services offered within the healthcare system [5].

### Dispensing errors—global, regional, and national perspective

In a study in Jamaica [https://www.carpin.org/events08/3rdScConf/4-2_MedicalErrors.pdf], it was noted that dispensing errors made up a significant proportion of the medication errors that occurred during a 6-week observation period. The causes reported for medication errors were illegible handwriting and confusing drug names. Use of advanced technology was anticipated to change the dispensing practices through writing electronic prescriptions with the hope of changing the pharmacy and healthcare landscape [6].

In order to reduce medication errors, a CareFusion Medication Management System was implemented in the Bahamas in 2015. CareFusion system is an intelligent medication management system that helps patients receive their medications quickly and effectively. Based on the demographics of the patient, the system also assists medical personnel in administering the right medication on the basis of medical history, age, and vital information of patients. The system helps in cost saving by providing correct medication to patients [7]. It was reported that the new medicine management system implemented at the Princess Margaret Hospital in the Bahamas led to increased efficiency of the pharmacists and reduced the occurrence of medication errors [7]. While Thomas et al. found that lack of sufficient number of pharmacists during peak hours was one of the major causes of dispensing errors [8], Kistner et al. found no correlation between work volume and dispensing errors [9]. A study by Ashcroft et al. recommended identifying incidence and nature of dispensing errors in order to design risk management strategies [10]. Even in the USA, at the national level, dispensing errors were observed by Flynn et al. in their study [11]. National Coordinating Council for Medication Error Reporting and Prevention (NCC-MERP) in its document titled “Taxonomy of Medication Errors” provides standard taxonomy to be used for assisting in recording and tracking of medication errors [12].

Few studies are available on dispensing errors in Trinidad and Tobago. In order to bridge this knowledge gap, the current research study was undertaken. The objective of the study was to identify reasons for dispensing errors at Eric Williams Medical Sciences Complex (EWMSC) in Trinidad and Tobago.

## Methodology

### Study design

An observational study was conducted for 2 weeks during May-June 2017, at the adult in- and outpatient as well as the pediatric pharmacy dispensaries of the Eric Williams Medical Sciences Complex (EWMSC) from Wednesday 31 May 2017, to Tuesday 13 June 2017. The observations were recorded during 7:00 am to 3:00 pm during the weekdays.

In the billing area, the investigators evaluated the prescriptions received by the pharmacists before and after they were billed to identify dispensing errors. The errors which were found after the billing of the prescription were recorded as dispensing errors. Patients’ prescription forms, incident forms, and pharmacy bills were viewed in order to compare the prescribed and dispensed medication for patients for the identification of any dispensing errors.

Following the filling of the prescription by the pharmacists, the prescriptions and medications were checked in the dispensing area. If an error was found while dispensing the medications to the patient, the investigator would intervene and inform the pharmacist about the error.

Similarly, in the cases of inpatient medication dispensing, the medications that were received in the wards were analyzed by going to the respective wards and verification was carried out to ascertain whether medications dispensed to the wards were correct.

### Study population

Individuals from the general population who visited the pharmacies at the EWMSC to receive medication either for themselves, a family member, relative, friend, or an inpatient were included in the study.

### Study sample

The study sample refers to the number of patients who visited the pharmacies within the study period of 7:00 am to 3:00 pm each day during the weekday from May 31, 2017 to June 13, 2017.

### Sample size

The sample size consisted of persons who attended the pharmacy (adult inpatient/outpatient and pediatric wards) with valid prescriptions and had at least one (1) item dispensed to them. For this study period, a total of 5154 filled prescriptions were analyzed.

### Data collection

Dispensing errors to be observed were identified and listed in tabular format. This was based on a previous study done by Beso et al. in an international setting [13]. A pilot study using the tool was carried out. After the pilot study, face and content validity were established with the help of experts in the field. During the study, occurrence of errors was noted with its time of occurrence and probable cause.

### Ethical approval

Ethical approval was obtained from the North Central Regional Health Authority (NCRHA) which governs the facility of EWMSC. Upon ethical approval from the NCRHA, the pharmacy manager was informed and the study was carried out.

### Data analysis

The details of any dispensing errors which occurred at any pharmacy of the EWMSC were recorded in a form and were assessed for the following parameters:

- The type of dispensing errors
- The rate at which the error occurred
- The impact of environmental factors such as time, area, or pharmacy type on the dispensing errors

If any dispensing errors occurred, they were classified according to the contents of Table 1 in the Appendix.

### Data protection—patient confidentiality

As data was being collected, neither the names nor personal details of the patients or those of the pharmacists were recorded at any stage of the data collection process.

## Results

**Fig. 1:**
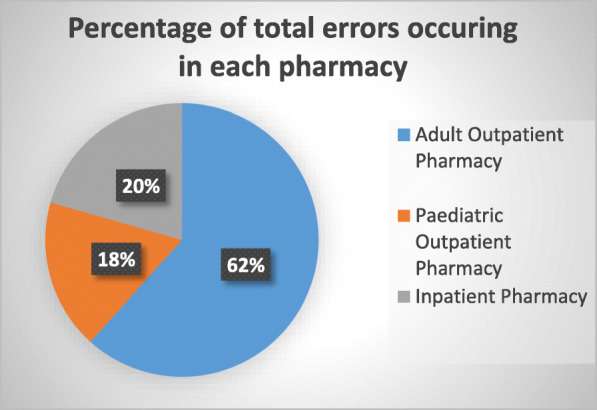
Relative composition of the total dispensing errors

**Fig. 2:**
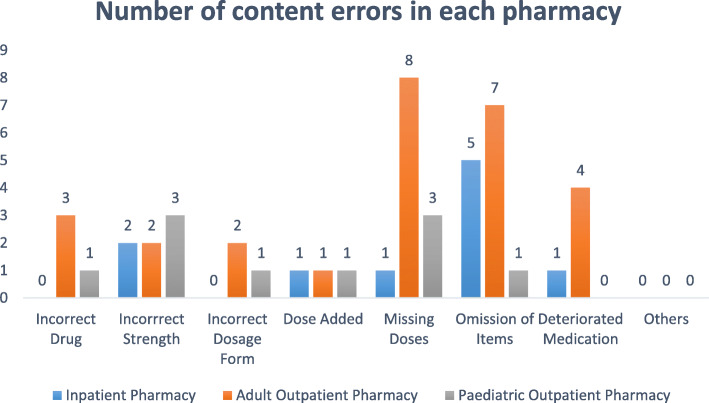
Distribution of content errors observed in each pharmacy at the EWMSC

**Fig. 3:**
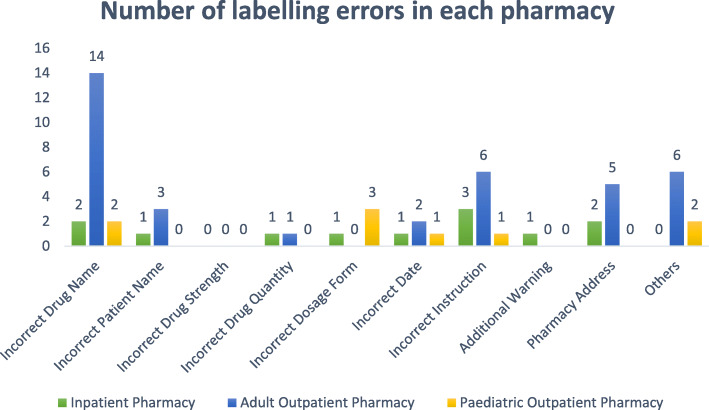
Labeling errors observed in each pharmacy at the EWMSC

**Fig. 4:**
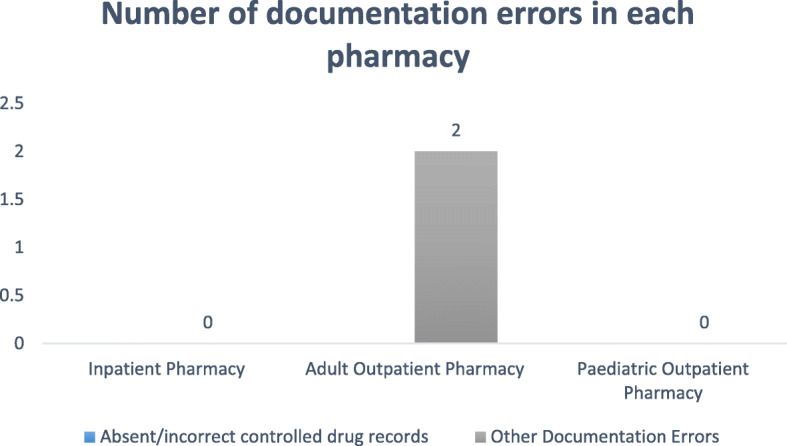
Documentation errors observed in each pharmacy at the EWMSC

**Fig. 5:**
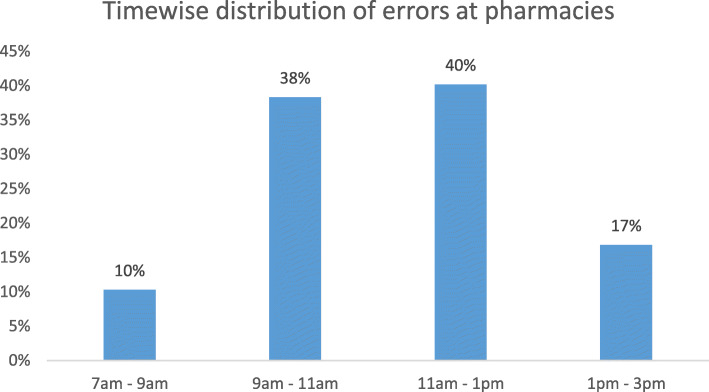
Time-wise distribution of the dispensing errors

During the 2-week study period, the pharmacies of the EWMSC dispensed 5154 prescriptions, which includes 2540 prescriptions from the adult outpatient pharmacy, 827 from the pediatric outpatient pharmacy, and 1987 from the inpatient pharmacy (Figs. 1 and 2). Of these prescriptions, a total of 109 dispensing errors were recorded during the study period. In some cases, more than one error was recorded for a single prescription (Fig. 3). A total of 68 errors occurring within the adult outpatient pharmacy, 19 occurring at the pediatric outpatient pharmacy, and 22 at the inpatient pharmacy were recorded (Fig. 4). Forty percent of the total errors occurred during 11 am-1 pm, 39% between 9 am and 11 am, 17% between 1 pm and 3 pm, and 10% between 7 am and 9 am (Fig. 5).

While pharmacists of the EWMSC acknowledged medication errors at the pharmacy, no records of dispensing errors were kept. The log book that was referred during the study had records only of prescription errors made by doctors.

## Discussion

### Frequency and types of errors

Several dispensing errors were observed at the pharmacies. These errors account for about 2.1% of all prescriptions filled at the complex. It is possible that the higher rate of dispensing errors observed at the EWMSC is due to the design of the study since pharmacists were not informed about the objectives of the study to avoid influence on the outcome of the study as well as to reduce bias. Both labeling and content errors were significant, unlike documentation errors that were negligible. The most commonly observed dispensing errors include missing doses, omission of items, incorrect patient name, and incorrect drug name. The percentage of errors to total prescriptions filled was 1.2% in in-patient pharmacy, 2.3% for the pediatric pharmacy, and 2.6% for the adult outpatient pharmacy.

### Potential causes of errors

High workload was evident, especially in the adult outpatient pharmacy which saw an average of 254 patients within a span of 8 h while a maximum of only 4 pharmacists were involved in the dispensing process at any given time. The 8-h time does not include lunch breaks where the number of patients waiting is at its peak, and the number of pharmacists dispensing medications is less. The influx of patients was found to be significantly high at times where over 100 patients were waiting to get their prescriptions filled. There was no substitution of pharmacists who were unavailable during the lunch break which increases the workload and stress on those pharmacists who were discharging their duty during this peak time. This could have led to higher dispensing errors. The high amount of errors can be corroborated with the time at which they occurred which is between 9 am to 1 pm. During 9 am to 1 pm usually a high number of patients visit doctors’ clinics and this high number of patients increases the work pressure on pharmacists while dispensing medications and filling the prescriptions. It was observed that the small number of pharmacists present at the outpatient pharmacy resulted in a buildup of patients and as the limited number of pharmacists try to tend to all waiting patients, they are more likely to cause dispensing error. During this time period of 9 am to 1 pm, in-patient pharmacy is extremely busy as doctors complete their ward rounds and prescribe required medicines for their patients. Other potential causes of dispensing errors were the doctors’ poor handwriting, similar drug names, and there were even occurrences where pharmacists were distracted.

Other plausible causes of dispensing error include failure of a few pharmacists to request identification form from the patient to verify their identity prior to dispensing. Incorrect patient names, spelling of names and incorrect personal details were recorded in the pharmacy database on a few occasions. This incorrect information would then be utilized on subsequent visits leading to potential dispensing errors. There were cases where additional medication was given because pharmacists did not make a proper note of drugs already dispensed on a prior visit. In case of the adult outpatient pharmacy, it was observed that the facility is only partially air conditioned and the fans being utilized did not function properly. This leads to uncomfortable working conditions for pharmacists while discharging their duties. The pharmacists usually complained of the heat and such discomfort during working hours contribute toward dispensing errors.

### Preventing dispensing errors

Some of the measures, both preventive and corrective, can lead to reduction in number of dispensing errors at pharmacy. Firstly, with respect to the adult outpatient pharmacy, an increase in the number of pharmacists at prescription as well as dispensing window can increase patient throughput and reduce workload on individual pharmacists. This may also help reduce stress among pharmacists leading to a decrease in stress-related errors. It was also noted that of a total of 8 pharmacy windows, only 4 were operational at a given point in time leading to underutilization of facility. Non-operational pharmacy windows could be made operational by adding more pharmacists to the existing strength.

Secondly, to reduce errors pertaining to incorrect drug/dosage dispensed, doctors need to be educated of the possible negative consequences of poor handwriting. Many drug names are similar, and hence, poor handwriting may lead to dispensing of wrong medication to the patient having potentially fatal outcomes. To avoid such situation, stock boxes and stock shelves should highlight differences between drugs with similar-sounding names. This practice could be applied in all pharmacies.

Thirdly, a periodic stock check would aid in eliminating omission and dosage-related errors. It was observed that pharmacists relied on memory to inform patients on the availability of medicines. On a few occasions, it was found that pharmacists inadvertently informed patients that the medicine prescribed was not in stock, was low in stock (thus a lower dosage was given), or strength prescribed was not available. This cascaded into patients being recommended to buy the drug privately or check other public pharmacies for the prescribed drug. Hence, periodic stock checking must be implemented.

Fourthly, in order to eliminate labeling errors, drug names should be double checked with the stock and patient names should be referenced to their national ID cards. It was observed that some pharmacists do reference the patient’s ID card when taking details and filling the prescriptions but some do not even ask for the patient’s ID card during the patient-pharmacist interaction. The double checking of the labels is easily applicable in in-patient pharmacy since prescriptions that are filled to be taken to the wards can be cross verified. However, in case of outpatient pharmacy, additional staff would help ease the process of dispensing while reducing medication errors and ensure smooth functioning.

Finally, it was observed that working conditions caused discomfort to pharmacists as the facility was not fully air conditioned. The complaints about the hot environment by pharmacists working at the dispensing area were observed as well as the actual conditions itself were felt by researchers. Fans did not function properly and some were even placed in such a way causing inconvenient working condition and a potential for a tripping hazard. It is required that pharmacists are provided comfortable working environment to avoid dispensing errors.

This study had a few limitations. (1) The logbook kept at the pharmacy only detailed prescribing errors made by doctors. (2) The nature of the study required it to be disguised so as to avoid bias in their functioning due to the presence of the observer. Although this information was known only to the pharmacy manager and the heads of the different pharmacies, the general staff were able to sense the purpose of the study, which might have influenced the outcome. (3) The study required investigators to visit individual wards regularly to determine the occurrence of dispensing errors from the in-patient pharmacy. The limited number of researchers made this difficult to visit wards regularly within the time constraints of the study. (4) The time period to conduct the study may have been too short to present a truly accurate reflection of the problem being studied.

## Conclusions

Dispensing errors were encountered in filling 2.1% of the prescriptions brought to pharmacies of the EWMSC, the majority of these included missing doses, omission of items, incorrect patient name, and incorrect drug name among many other errors. The factors which influenced these dispensing errors include but are not limited to heavy workload, distractions, failure to verify patient information, and uncomfortable working conditions. Efforts should be made to minimize the stress on the pharmacists, enforce protocol for patient identification and verification of items dispensed.

## Data Availability

All data generated or analyzed during this study are included in the article.

## Appendix

**Table 1:** Types of dispensing errors and their total occurrence

Type of error	Total number of errors			
	Inpatient	Adult outpatient	Pediatric outpatient	Total
**Content errors**				
Incorrect drug	0	3	1	4
Incorrect strength	2	2	3	7
Incorrect dosage form	0	2	1	3
Dose added	1	1	1	3
Missing doses	1	8	3	12
Omission of items	5	7	1	13
Deteriorated medication	1	4	0	5
Other content errors	0	2	0	2
**Total content errors**	**10**	**29**	**10**	**49**
**Labeling errors**				
Incorrect patient name	2	14	2	18
Incorrect drug name	1	3	0	4
Incorrect drug strength	0	0	0	0
Incorrect drug quantity	1	1	0	2
Incorrect dosage form	1	0	3	4
Incorrect date	1	2	1	4
Incorrect instruction(s)	3	6	1	10
Additional warning	1	0	0	1
Pharmacy address	2	5	0	7
Other labeling errors	0	6	2	8
**Total labeling errors**	**12**	**37**	**9**	**58**
**Documentation errors**				
Absent/incorrect controlled drug records	0	0	0	0
Other documentation error	0	2	0	2
**Total documentation errors**	**0**	**2**	**0**	**2**
**Total errors**	**22**	**68**	**19**	**109**

## Abbreviations

EWMSC: Eric Williams Medical Sciences Complex
NCC-MERP: National Coordinating Council for Medication Error Reporting and Prevention
NCRHA: North Central Regional Health Authority
